# Pain is vital in resuscitation in trauma

**DOI:** 10.1051/sicotj/2019028

**Published:** 2019-08-15

**Authors:** Theodosios Saranteas, Andreas Kostroglou, Dimitrios Anagnostopoulos, Dimitrios Giannoulis, Pantelis Vasiliou, Andreas F. Mavrogenis

**Affiliations:** 1 Second Department of Anesthesiology, National and Kapodistrian University of Athens, School of Medicine, ATTIKON University Hospital Athens Greece; 2 Fourth Department of Surgery, National and Kapodistrian University of Athens, School of Medicine, ATTIKON University Hospital Athens Greece; 3 First Department of Orthopaedics, National and Kapodistrian University of Athens, School of Medicine, ATTIKON University Hospital Athens Greece

**Keywords:** Trauma, Pain, ATLS, Anesthesia, Fractures

## Abstract

Implementation of the ATLS algorithm has remarkably improved the resuscitation of trauma patients and has significantly contributed to the systematic management of multi-trauma patients. However, pain remains the most prevalent complaint in trauma patients, and can induce severe complications, further deterioration of health, and death of the patient. Providing appropriate and timely pain management to these patients prompts early healing, reduces stress response, shortens hospital Length of Stay (LOS), diminishes chronic pain, and ultimately reduces morbidity and mortality. Pain has been proposed to be evaluated as the fifth vital sign and be recorded in the vital sign charts in order to emphasize the importance of pain on short- and long-term outcomes of the patients. However, although the quality of pain treatment seems to be improving we believe that pain has been underestimated in trauma. This article aims to provide evidence for the importance of pain in trauma, to support its management in the emergency setting and the acute phase of patients’ resuscitation, and to emphasize on the necessity to introduce the letter P (pain) in the ATLS alphabet.

## Introduction

Originating in ancient Greek medical terminology, the word “trauma” illustrates a physical injury, a wound, or a defeat. Then, combat trauma was significant and well represented in the literature of the Ancient Greeks. Nowadays, trauma constitutes the leading cause of death in people younger than 44 years old and the leading source of disability in the laboring age population [1–3], being responsible for at least 10% of overall deaths [1]. Implementation of the ATLS algorithm has remarkably improved the resuscitation of trauma patients and, from an educational standpoint it has significantly contributed to the systematic management of multi-trauma patients [1–3]. However, pain remains the most prevalent complaint in trauma patients, and can induce severe complications, further deterioration of health and death to the patient. Providing appropriate and timely pain management to these patients prompts early healing, reduces stress response, shortens hospital Length Of Stay (LOS), diminishes chronic pain, and ultimately reduces morbidity and mortality [4].

Pain has been proposed to be evaluated as the fifth vital sign and is recorded in the vital sign chart [5], in order to emphasize the importance of pain on short- and long-term outcomes of the patients. However, we believe that pain has been underestimated in trauma [6, 7], yet the quality of pain treatment seems to be improving [8]. As a result, “oligoanalgesia” is quite common in injured patients in prehospital and emergency medicine, hence many efforts have been focused on improving pain management in trauma setting [8–10]. More importantly, inadequate analgesia is more frequent in the substance abuse trauma patients in whom standard intravenous medications, including opioids, are unsatisfactory [11]. This article aims to provide evidence for the importance of pain in trauma, to support its management in the emergency setting and the acute phase of patients’ resuscitation, and to emphasize on the necessity to introduce the letter P (pain) in the ATLS alphabet.

## The ABCDE algorithm

The management of trauma patients mandates a prompt survey and rapid implementation of targeted interventions [12]. Trauma patients should be reevaluated as frequently as possible in order to identify any clinical deterioration that may prove fatal. The ABCDE systematic approach is an immediate and definitive assessment of a trauma patient; using that algorithm, physicians may be able to recognize life-threatening injuries and to prioritize their interventions accordingly. The letters A (airway), B (breathing), and C (circulation) are the pylons of the ATLS algorithm indicating that patients’ oxygenation and hemodynamic stabilization are invariably the ultimate priority when assessing and treating trauma patients with life-threatening injuries. Additionally, the letters D (disability) and E (exposure) possess a significant role in the ATLS algorithm, through which a basic neurological evaluation as well as a whole body inspection can be thoroughly achieved.

## Pain: an underestimated vital symptom in trauma

Pain is the most common complaint among trauma patients in the Emergency Room (ER) [4]. It is also widely acknowledged that the vast majority of patients evaluate their pain treatment insufficient, frequently reporting low satisfaction scores [4]. Under-treated pain may be misunderstood by the patient as indifference on the physicians’ part, thus compromising patients’ ability to interact and comply with the clinical environment and to cooperate with physicians’ instructions [13]. Despite that, there is lack of implemented evidence-based protocols for adequate pain management in trauma patients [14], indicating the complexity of the issue. Although certain efforts are always put in by the ER staff to alleviate pain in trauma patients, the way they approach pain is often inconsistent and, in some cases, very superficial. More specifically, it has been documented that pain score scales are underutilized [15], while pain killers are given without detailed evaluation of pain symptoms; therefore, pain intensity is either downrated or overestimated, leading to suboptimal or overzealous pain treatment.

A recent study reported that pain management in trauma patients varies significantly among emergency healthcare providers, highlighting the importance of consistent pain registration in patients’ files [16]. More to the point, physicians properly educated in the ATLS algorithm are exclusively concentrated on managing life-threatening conditions, thus (especially in unstable patients) often delay in the assessment and management of pain [17]. A graphic example is the retrospective study of Bakkelund et al. [18], where it came out that trauma patients, despite their higher pain scores, were treated with the same doses of morphine as patients with chest pain, in prehospital care. Remarkably, a previous study revealed that one third of trauma patients are not assessed for pain until they are discharged from the ER [17]. Having perceived the problem of pain mismanagement in the ER, Berben et al. [6] investigated and delineated the specific barriers of optimal pain management in trauma patients; five chief reasons were pointed out and analyzed as follows: (a) inability of accurate estimation of pain intensity, (b) frequently downplayed importance of pain severity by the majority of ER physicians, (c) inadequate interdisciplinary feedback among ER physicians and pain specialists (lack of an acute pain service teams), (d) organizational problems hindering and impeding the prompt assessment of pain intensity and the administration of proper pain killers, and (e) factors pertaining to patients’ personality and attitude (patients’ refusal of pharmacological pain treatment) [6].

Cohen et al. [19] targeted the heart of the problem which is the fear of ER physicians both to dispense and to administrate opioids in proper dosing regimens. Respiratory depression, cardiovascular instability and gastric content aspiration, common side effects of opioids, are the main reasons for the reluctance and unwillingness of ER physicians to provide narcotics to trauma patients in the emergency setting [15, 19]. Respiratory depression may induce hypoventilation and hypercapnia, increasing the cerebral blood flow and the intracranial pressure, aggravating a possible secondary trauma to the brain tissue [20]. In addition, sedation in a traumatized patient with a compromised mental status or drug abuse may jeopardize the airway patency, leading to detrimental complications. The hesitancy of opioid administration is more apparent in physicians with less experience in pain management [9]. Therefore, concrete evidence of education deficits in pain management among ER physicians is obvious. In that way, the revolutionization of the current practice through incorporating the letter P (pain) in the ATLS algorithm is inflicted more than ever before.

However, assessment of trauma patients is difficult, and attention should be paid to specific occasions that are complicated in origin. For instance, patients with alcohol or drug abuse, polytrauma, severe head injury or limited level of consciousness, and elderly trauma patients may not be able to provide helpful information [4, 17, 21]. Pain management in patients with chronic opioid use may be extremely significant [22]. Tolerance to opioids and potential opioid withdrawal, on top of acute pain symptoms, require increased doses of opioids; in these cases, appropriate opioid titration to alleviate pain becomes a downright challenge [22]. Additionally, an interdisciplinary approach and cooperation with other teams exhibiting higher expertise in pain management is essential. Nevertheless, as challenging and complicated as pain management may seem, it should be emphasized and deeply comprehended that pain relief improves the outcome of trauma patients. Therefore, accurate pain assessment and treatment should not only be deliberated a priority but an obligatory necessity and human right for all trauma patients suffering from acute pain.

## Stress response to pain

Stress response is a sequence of changes, which occurs in neuroendocrine, immune, and metabolic systems [23]. With regard to the evolution under difficult environmental conditions, stress response benefits the survival by maintaining the homeostasis of the organism [24–26]. Stress response associated with injury of any category has been studied more in surgical patients, but the same features have been noticed in trauma patients as well [25]. In general, it could be stated that the magnitude of stress response is proportional to the severity of the injury, it being significantly more intense in polytrauma patients than in surgical population [19].

Stress response is amplified by the presence of pain, evoking a sequence of alterations in neuroendocrine system [27]. Pain stimuli stemming from injured tissues activate the Hypothalamic-Pituitary-Adrenal (HPA) axis and trigger the autonomous Sympathetic Nervous System (SNS). Consequently, pituitary hormones are excessively released and the production of catecholamines in the adrenal medulla tops out immediately after trauma. Overall, there is an excessive secretion of catabolic hormones (catecholamines, cortisol) and at the same time suppression of anabolic hormones (insulin) [25]. Therefore, the enhanced levels of catecholamines in serum and the augmentation of the SNS tone can generate severe complications in multiple systems.

### Cardiovascular system

High levels of catecholamines and SNS activation have been repeatedly highlighted by the literature for their deleterious effects in the cardiovascular system [28]. Arrythmiogenic effects, hypertension, and tachycardia predominate, which under certain pathological substrates may lead to myocardial ischemia and infarct [23]. The latter is crucial for patients who suffer from coronary artery disease or cardiac failure and present diminished cardiovascular reserves.

### Neuroendocrine and metabolic system

Elevated catecholamines and SNS activation modulate multilaterally the neuroendocrine function and metabolic status. ACTH, which is produced by the anterior pituitary, stimulates glucocorticoid secretion by the adrenal cortex. Cortisol then promotes catabolism and gluconeogenesis, inhibits the uptake of glucose by the cells (a pathological condition known as insulin resistance) [28], and disrupts the immune process. In stress free conditions, the elevated concentrations of cortisol additionally suppress the secretion of ACTH through a negative feedback mechanism. However, this negative feedback process is disrupted after surgery [28] and consequently the same probably occurs in a major trauma.

### Immune system

A prompt response to painful stimuli activates the production of proinflammatory cytokines through immunoactive cell activation at the site of the injury [27]. The latter plays a substantial role in wound healing, especially in its early stages [24, 29]. Activation of HPA and SNS disrupts the production of cytokines, thus leading to dysregulation of the healing process [24]. Furthermore, the circulating catecholamines and the increased cortisol secretion enhance the production of glucose. Indeed, the subsequent hyperglycemia has been shown to induce a well-documented negative effect in wound healing [28, 30, 31]. Immunosuppression in addition to dysfunction of the coagulation mechanisms makes peripheral tissues more vulnerable to wound infections.

### Coagulation

The SNS and the HPA axis prompt vasoconstriction, hemoconcentration, and endothelial dysfunction, while it enhances acute inflammatory tissue reactions [32]. In addition, activation of platelets and blood viscosity develops [26]. The net result is hypercoagulation that may result in detrimental cardiovascular events such as deep vein thrombosis and pulmonary embolism, especially in patients with underlying high thrombotic risks such as coronary heart disease and thrombophilia.

## The paradigm of pain in thoracic trauma

Thoracic trauma constitutes the most studied field in trauma with respect to trauma-related pain and consequences [33–39]. In thoracic trauma, not only do pain fallouts affect patients’ morbidity but principally their mortality more than any other type of injury. The high morbidity and mortality in trauma patients are mainly associated with respiratory system impairment. Thoracic pain prevents patients from breathing sufficiently, leading to retention of secretions, atelectasis, pneumonia, and respiratory failure [33–36]. With aggressive pain treatment, effortless breathing, efficient coughing, effective and intense physiotherapy can be successfully implemented. Justifiably, therefore, pain alleviation in thoracic trauma should be reckoned of vital and fundamental importance, and pain management in these patients should be considered one of the pillars of thoracic trauma treatment.

However, the best analgesic technique in thoracic trauma is unclear. Manay et al. [37] reported that Thoracic Epidural Analgesia (TEA) halved the number of days that the patients were intubated and significantly decreased the incidence of pneumonia. In a multicenter cohort study, Gage et al. [38] concluded that TEA reduced mortality in patients with three or more rib fractures. Conversely, in trauma patients with rib fractures, a recent meta-analysis of RCTs that compared TEA with other analgesic regimens, exhibited that TEA did not reduce the mortality, need for ventilation or LOS both in the ICU and the hospital overall [39]. Further analysis of this study has shown, however, that methodology weaknesses did not allow the superiority of TEA over other pain therapy remedies to be revealed. Two other studies [40, 41] have demonstrated that TEA resulted in significant reduction in the duration of mechanical ventilation when compared to parenteral opioids. Consequently, although a concrete conclusion about the best analgesic technique is still questionable, aggressive pain treatment is considered indispensable in thoracic trauma patients [42].

To optimize pain management, the implementation of the so-called multimodal analgesia that is defined as the utilization of a variation of medications and analgesic techniques, acting with different mechanisms and at multiple sites in the central and/or peripheral nervous system is well established [43]. This combination not only results in synergistic effect of the analgesic regimens, but also it does reduce substantially the side effects of each drug or analgesic technique, due to the fact that the required dosages of each medication are lower [44]. The multimodal regimen could be described schematically as a pyramid configuration, where regional analgesia comprises the basis, while opioid and non-opioid pain relievers constitute the sides resting upon [42].

## Pain as the fifth vital sign

Pain has been proposed to be evaluated as the fifth vital sign [5, 13, 45]. However, heavily influenced by the patient’s psychological status (anxiety and depression), evaluation of pain cannot be objective. Additionally, pain cannot be considered a clinical sign such as the existing vital signs (blood pressure, heart rate, respiratory rate, temperature) but rather a subjective clinical symptom. However, pain may alter vital signs and occasionally, such as in thoracic trauma it may be related to increased mortality.

In the event of acute pain, it is considered a proper approach to carefully appraise patients based on OPQRST. OPQRST is the acronym of Onset of the event, Provocation or palliation, Quality of the pain, Region and radiation, Severity, and Time (history). The aforementioned factors are important while examining patients upon admission, and the Visual Analog Scale (VAS) and Verbal Rating Scale (VRS) are assessed in order the severity of pain is understood [46, 47]. Unidimensional pain scores such as the VAS and the Numerical Rating Scale (NRS) have proved to be valid and reliable for treatment evaluation, but they do not estimate pain comprehensively [48–50]; nevertheless, single dimension scales contribute to a higher quality of pain treatment [51]. Dynamic (associated with pain-related complications) and static (representing the patient’s comfort) components of pain must be assessed and invariably reappraised as the other vital signs and letters of the ATLS algorithm. For instance, an acute deterioration of pain intensity or change of its features and characteristics may contribute to prompt diagnosis of an underlying clinical complication such as the compartment syndrome.

Patients’ discomfort due to severe pain can also influence the responsiveness of physicians to difficult situations. Inexperienced physicians who are flustered and flurried in emergency settings, lose rapidly and irretrievably the confidence of their patients. A vicious cycle, therefore, is initiated that may lead to uncontrolled reactions from both sides. For this reason, not only does effective and thorough pain treatment guard doctors against patients’ emotional instability but it also cements their practice, in that, they will be able to interact with the medical environment through resilience and calm in the face of any adversity. On the other hand, definitely, any measures to promptly identify and treat life-threatening conditions, to preserve life and avoid secondary injury take priority in the management of trauma patients. Pain is important and vital to be managed; however, under no circumstances should interventions toward pain alleviation hinder the resuscitation process. Therefore, the letter P should be taken into account at the end of a successful resuscitation and at the beginning of any post-resuscitation interventions; in that way, it could bridge the ATLS protocols and any intervention following resuscitation. Once the primary survey of the trauma patient is completed, the resuscitation of pain could be integrated in the secondary evaluation, where a thorough clinical examination is carried out; in that case, the management of pain in trauma could form an indispensable part of the ATLS algorithm.

## Conclusion

Pain evaluation and management from the prehospital/emergency settings up to patients’ discharge should be considered a basic principle and plank for patients’ psycho-physical integrity. Patients’ holistic care encompasses five vital signs, the fifth of which should be Pain. Very importantly, the letter P (pain) should be introduced to the ATLS algorithm, and pain management of the trauma patients should be converted from an absolute challenge into a daily practice in the context of emergency medicine.

## Conflicts of interest

All authors declare no conflicts of interest.
